# Non-carbonic buffer power of whole blood is increased in experimental metabolic acidosis: An *in-vitro* study

**DOI:** 10.3389/fphys.2022.1009378

**Published:** 2022-10-21

**Authors:** Martin Krbec, Petr Waldauf, Francesco Zadek, Serena Brusatori, Alberto Zanella, František Duška, Thomas Langer

**Affiliations:** ^1^ Department of Anaesthesia and Intensive Care Medicine, The Third Faculty of Medicine, Charles University and FNKV University Hospital, Prague, Czechia; ^2^ Department of Pathophysiology and Transplantation, University of Milan, Milan, Italy; ^3^ Department of Medicine and Surgery, University of Milan-Bicocca, Monza, Italy; ^4^ Department of Anesthesia, Critical Care and Emergency, Fondazione IRCCS Ca’ Granda Ospedale Maggiore Policlinico, Milan, Italy; ^5^ Department of Anesthesia and Intensive Care Medicine, Niguarda Ca’ Granda, Milan, Italy

**Keywords:** acid-base equilibrium, buffers, blood, metabolic acidosis, blood-gas analysis, base excess

## Abstract

Non-carbonic buffer power (β_NC_) of blood is a pivotal concept in acid-base physiology as it is employed in several acid-base evaluation techniques, including the Davenport nomogram and the Van Slyke equation used for Base excess estimation in blood. So far, β_NC_ has been assumed to be independent of metabolic acid-base status of blood, despite theoretical rationale for the contrary. In the current study, we used CO_2_ tonometry to assess β_NC_ in blood samples from 10 healthy volunteers, simultaneously analyzing the electrolyte shifts across the red blood cell membrane as these shifts translate the action of intracellular non-carbonic buffers to plasma. The β_NC_ of the blood was re-evaluated after experimental induction of metabolic acidosis obtained by adding a moderate or high amount of either hydrochloric or lactic acid to the samples. Moreover, the impact of β_NC_ and pCO_2_ on the Base excess of blood was examined. In the control samples, β_NC_ was 28.0 ± 2.5 mmol/L. In contrast to the traditional assumptions, our data showed that β_NC_ rose by 0.36 mmol/L for each 1 mEq/l reduction in plasma strong ion difference (*p* < 0.0001) and was independent of the acid used. This could serve as a protective mechanism that increases the resilience of blood to the combination of metabolic and respiratory acidosis. Sodium and chloride were the only electrolytes whose plasma concentration changed relevantly during CO_2_ titration. Although no significant difference was found between the electrolyte shifts in the two types of acidosis, we observed a slightly higher rate of chloride change in hyperchloremic acidosis, while the variation of sodium was more pronounced in lactic acidosis. Lastly, we found that the rise of β_NC_ in metabolic acidosis did not induce a clinically relevant bias in the calculation of Base excess of blood and confirmed that the Base excess of blood was little affected by a wide range of pCO_2_.

## Introduction

In human blood, two classes of buffers limit pH fluctuations in response to an acid load: the bicarbonate buffer system and the non-carbonic buffers. The latter consist of plasma proteins, phosphate, and, most importantly, hemoglobin inside the red blood cells—RBC (Van Slyke, 1922). While both classes of buffers act in metabolic acid-base disorders, only the non-carbonic buffers limit pH changes in respiratory derangements (Giebisch et al., 1955). To illustrate this fact, consider a case of respiratory acidosis, where the accumulation of CO_2_ shifts the H_2_CO_3_/HCO_3_^−^ equilibrium towards HCO_3_^−^ production and H^+^ release. It is evident that HCO_3_^−^ cannot buffer H^+^ released in this context. Consequently, as bicarbonate does not play a role in buffering of respiratory disorders, the ability of blood to resist respiratory derangements should not be altered in metabolic acidosis, a condition that is characterized by a reduced concentration of bicarbonate but preserved concentration of non-carbonic buffers.

The principle of independence between [HCO_3_^−^] and non-carbonic buffering is used in the traditional Davenport [HCO_3_^−^]/pH diagram (Davenport, 1958). In this diagram, two classes of isopleths can be observed: ascending convex curves representing titration by a strong acid or base at fixed pCO_2_, and descending lines representing titration by CO_2_ at a fixed metabolic acid-base status. Notable characteristics of the latter are that for a given composition of non-carbonic buffers, that determines their slope, they are all linear and parallel; and that their displacement along the [HCO_3_^−^] axis corresponds to the metabolic acid-base status of the sample. However, Siggaard-Andersen has shown experimentally that these lines are, in fact, convex curves, and theoretically, they converge towards the acid side. Despite that, he concluded that “as an approximation, we can assume the pH, [HCO_3_^−^] equilibration curves to be linear and parallel and the slope to vary linearly with hemoglobin concentration” (Siggaard-Andersen, 1974).

Nowadays, the Base excess of whole blood [BE(B)], calculated with the Van Slyke equation, is used to assess the metabolic acid-base status of blood (Siggaard-Andersen, 1977; CLSI, 2009). This equation includes the non-carbonic buffer power of blood (β_NC_), which represents the slope of the [HCO_3_^−^]/pH curve during CO_2_ equilibration (Van Slyke, 1922; Brown and Clancy, 1965; Michel et al., 1966). The assumption of no interaction between metabolic acid-base disorders, that alter [HCO_3_^−^], and β_NC_ is reflected in the Van Slyke equation by the fact that β_NC_ depends only on hemoglobin concentration (Hb, expressed in g/dl):

$$\beta_{NC}=1.43\times Hb+7.7$$ (1)

β_NC_ is used together with the actual bicarbonate of the sample ([HCO_3_^−^]_act_, expressed in mmol/L) to calculate plasma [HCO_3_^−^] that would be present if pH of 7.4 were achieved by altering pCO_2_. This value is known as Van Slyke standard bicarbonate—VSSB (Siggaard-Andersen, 1974; Wooten, 2003):

$$VSSB={\left[{HCO}_{3}^{-}\right]}_{act}+\beta_{NC}\times \left(pH-7.4\right)$$ (2)

At the pH of 7.4, the proportion of dissociated non-carbonic buffers is constant as determined by the difference between pK_a_ of each buffer and pH. Therefore, any difference between VSSB and the normal [HCO_3_^−^] of 24.4 mmol/L can only be explained by the presence of a nonvolatile acid or base. Finally, this difference is multiplied by a factor that compensates for the fact that at the pH of 7.4 [HCO_3_^−^] inside RBC changes slightly less than in plasma:

BE(B)=(1−0.014×Hb)×(VSSB−24.4) (3)

Manipulation of pCO_2_ in blood is accompanied by electrolyte shifts across the RBC membrane, a phenomenon known as the chloride shift or Hamburger effect (Warburg, 1922; Van Slyke et al., 1923; Giebisch et al., 1955; Langer et al., 2015). It can be shown (see Supplementary Text Part I) that these shifts are an essential condition that allows the intracellular buffers to act upon plasma, and can, therefore, be regarded as a proxy for the action of the intracellular buffers.

The experiments investigating the effect of metabolic acid-base status on β_NC_ (Siggaard-Andersen, 1974) were carried out before the development of direct pCO_2_ electrodes (Astrup and Severinghaus, 1986). The technique of indirect pCO_2_ determination is lengthy and inevitably leads to some degree of error due to ongoing cell metabolism. Moreover, it allowed β_NC_ calculation from only few points of pH/[HCO_3_^−^] curve. Nowadays, the acid-base analyzers allow determining β_NC_ with higher precision and consistency. In light of this, we decided to perform this *in-vitro* study to 1) quantify β_NC_ under normal conditions and during experimental metabolic acidosis—hyperchloremic and lactic, 2) describe the associated electrolyte shifts, and 3) evaluate possible implications for the calculation of Base excess.

## Materials and methods

This *in-vitro* experimental study was carried out at the Department of Intensive Care of Policlinico di Milano, Italy. Ten volunteers with no record of hematologic or metabolic diseases were recruited from the Intensive Care Unit staff after administration of written informed consent. The number of enrolled subjects was chosen arbitrarily because no estimate of the expected change of β_NC_ was available to perform an *a-priori* power analysis.

### Blood collection and sample handling

Venous blood was sampled for laboratory analysis of complete blood count, albumin, and phosphate. Further 24 ml were collected in lithium-heparin tubes for β_NC_ analysis. This blood was transferred into a single syringe to ensure homogeneity, and a baseline blood-gas analysis was performed. Then, the blood was split into five 4-ml aliquots and cooled down to 4°C to halt cell metabolism and lactate production.

### Stock solutions

Five stock solutions were prepared to experimentally induce metabolic acidosis by increasing the strong ion difference (SID) of the blood aliquots and reducing their [HCO_3_^−^]. These solutions were prepared by combining normal saline, hydrochloric acid (HCl), L-(+)-lactic acid (HLac), sodium hydroxide, and distilled water. Their composition is reported in Supplementary Table S1. To minimize potential transmembrane shifts of water caused by perturbation of osmolarity, the solutions were tailored to have [Na^+^] similar to plasma. The stock solutions were designed in such a way that adding approximately 200 µl into 4 ml of blood should either:

1. Have a minimum impact on SID and [HCO_3_^−^]—control (Ctr)
2. Increase [Cl^−^], and decrease SID and [HCO_3_^−^] by 7.5 mEq/L—moderate hyperchloremia (Cl 7.5)
3. Increase [Lac^−^], and decrease SID and [HCO_3_^−^] by 7.5 mEq/L—moderate lactatemia (Lac 7.5)
4. Increase [Cl^−^], and decrease SID and [HCO_3_^−^] by 15 mEq/L—severe hyperchloremia (Cl 15)
5. Increase [Lac^−^], and decrease SID and [HCO_3_^−^] by 15 mEq/L—severe lactatemia (Lac 15)

As blood can be viewed as a two-compartment system, the expected plasma SID change is dependent on three factors: the volume and concentration of the acid added, the volume of the plasma phase, and the proportion of the acid that is transferred into RBC. In order to achieve the desired shift in plasma SID, we calculated the required volume of the stock solutions for each volunteer individually using the hemoglobin concentration from the initial blood gas analysis and the standard mean cell hemoglobin concentration of 33 g/dl. We assumed that 70% of the acid added remains in plasma as determined by a series of preliminary experiments (data not shown). All aliquots of each volunteer were diluted by the same volume, ensuring that all blood components that may possibly affect acid-base, such as hemoglobin and plasma proteins, were diluted equally.

### β_NC_ assessment

To measure β_NC_, we used the method of CO_2_ tonometry coupled with repetitive blood-gas analyses, as previously described elsewhere (Langer et al., 2021). Each aliquot was diluted with one of the stock solutions, drawn into a pretreated anti-foam syringe (T310, RNA Medical, United States), and placed into a CO_2_ tonometer (EQUILibrator, RNA Medical, United States) for 12 min, where they were heated to 37°C and oxygenated to >92% using custom-made gas mixtures containing 21% O_2_ and either 2% or 20% CO_2_. The same gas mixtures were then used to manipulate pCO_2_ of each aliquot in the range of 20–120 mmHg. Throughout the experiment, pH, pCO_2_, and concentration of main electrolytes were analyzed repeatedly using a point-of-care blood-gas analyzer (ABL90, Radiometer, Denmark), obtaining at least 10 data points for each aliquot.

The possible confounding effect of hemolysis, resulting from mechanical and thermal stress during the CO_2_ tonometry, was found to be negligible in preliminary experiments as the median percentage of hemolyzed RBC was less than 1% (for further details, see Supplementary Text Part II). Other confounders that commonly affect acid-base physiology (e.g., renal failure, administration of i.v. fluids, hypoalbuminemia, ketoacidosis, etc.) were excluded in our experimental setting.

### Definitions and calculations

The buffer power of a simple buffered solution is pH-dependent. Although this dependency is attenuated in the case of the complex buffer mixture present in human blood (Van Slyke, 1922), a representative pH value for performing the calculations had to be chosen. We decided to use the value of 7.2, rather than the more common 7.4, to guarantee that the analyses would be performed in a pH range explored during the experiments.

We used the following formula for estimation of SID (in mEq/l):

$$SID=\left[{Na}^{+}\right]+\left[K^{+}\right]+2\times \left[{Ca}^{2+}\right]-\left[{Cl}^{-}\right]-\left[{Lac}^{-}\right]$$ (4)

where the terms in square brackets denote the concentration (in mmol/L) of the appropriate ion or molecule. [Mg^2+^] was not included in the SID calculation as the blood gas analyzer did not directly measure it. The concentration of bicarbonate was calculated as:

$$\left[{HCO}_{3}^{-}\right]=S\times {pCO}_{2}\times 10^{pH-{pK}_{1}^{\prime }}$$ (5)

where the pCO_2_ stands for partial pressure of CO_2_ expressed in mmHg, S = 0.0307 mmol/L/mmHg represents CO_2_ solubility in plasma, and pK_1_′ = 6.095 represents the negative logarithm of the apparent first dissociation constant of carbonic acid in blood (CLSI, 2009).

To quantify the metabolic acid-base status of the blood aliquots, we defined SID_7.2_ as SID present when the pH of 7.2 is achieved by pCO_2_ manipulation. To cope with the fact that achieving a prespecified pH value is not possible during CO_2_ tonometry and to minimize the accumulation of measurement errors, SID_7.2_ was obtained by interpolation from all SID and pH pairs of the given aliquot. Accuracy of the collected data was assessed by correlating SID and [HCO_3_^−^] at the pH of 7.2 in the aliquots of each volunteer - see Supplementary Text Part III.

### β_NC_ and the electrolyte transfers

A second-order polynomial (to account for both the convexity and convergence of the curves) was fitted through the pH and [HCO_3_^−^] data points of each aliquot. The goodness of fit was assessed using the Root mean square error (RMSE). Curves with high RMSE defined using Tukey’s outlier detection method were excluded. β_NC_ was then calculated as the negative of the first derivative of the fitted polynomial at the pH of 7.2:

$$\beta_{NC}=-\frac{d\left[{HCO}_{3}^{-}\right]}{dpH}$$ (6)

The change of [HCO_3_^−^], and hence also β_NC_, is determined by the buffer action of plasma weak acids and the electrolyte shifts that transfer the action of intracellular buffers to plasma. To quantify these shifts, the process of curve fitting and goodness-of-fit assessment was repeated for pH vs. [Na^+^], [K^+^], [Cl^−^], and [Lac^−^]. For each electrolyte, the rate of change of its concentration in relation to pH was calculated by substituting d[Na^+^], d[K^+^], -d[Cl^−^], and -d[Lac^−^] for d[HCO_3_^−^] in Eq. 6. A representative value of β_Na_, β_K_, β_Cl_, and β_Lac_ was calculated for pH of 7.2.

### The effect on BE(B)

Our experiments allowed us to test three attributes crucial for BE(B) calculation and applicability: 1) the accuracy of β_NC_ derived from hemoglobin concentration, 2) the proportionality between the amount of acid added through the stock solutions and the fall of BE(B), and 3) the pCO_2_ invariance of BE(B).

First, the measured β_NC_ at the pH of 7.2 was compared with β_NC_ calculated using Eq. 1 in each group of aliquots. Second, BE(B) was calculated in all blood-gas analyses according to Eqs. 1–3. For each aliquot, a representative value at pCO_2_ of 40 mmHg [BE(B)_VanSlyke_] was obtained by interpolation from all BE(B) and pCO_2_ pairs due to the same reasons that were mentioned for SID_7.2_. This value was compared with the true change of Base excess [ΔBE(B)_true_] determined from the amount of acid added experimentally through the stock solutions:

$${\Delta BE\left(B\right)}_{true}=\frac{V_{dil}\times \Delta SID}{0.004+V_{dil}}$$ (7)

where V_dil_, expressed in liters, refers to the volume of the added stock solution, 0.004 L is the volume of blood aliquots, and ΔSID is the difference between SID of the stock solution used and the control stock solution (see Supplementary Table S1). Finally, the pCO_2_-induced variation of BE(B) was evaluated by plotting BE(B) of every single blood-gas analysis against pCO_2_. To account for substantial baseline differences, we used a normalized value [BE(B)_norm_] obtained by subtracting BE(B)_VanSlyke_ of the corresponding aliquot from individual BE(B) values.

### Statistical analysis

The data collected by the blood gas analyzer were exported into a spreadsheet that was handled in Microsoft Excel 365. Descriptive statistics, curve interpolation, and β calculation were done using GraphPad Prism 8.0.1. Analysis of the relationship between metabolic acid-base status of the aliquots and β_NC_ as well as β of individual electrolytes was performed using R software, version 4.2.0 (R Core Team, 2022; RStudio Team, 2022). We used a linear mixed effect model (LMEM) with random intercept (individual ID). SID_7.2_, acid type, and their interaction were considered fixed effects. The superiority of the model with or without the interaction between SID_7.2_ and acid type was evaluated by the likelihood-ratio test. The results are reported as mean ± SD or median [1Q—3Q], as appropriate. *p* value < 0.05 was considered statistically significant.

## Results

Ten volunteers (aged 30 ± 5 years, 5 females) were enrolled. Prior to any manipulation, blood hemoglobin was 14.6 ± 1.4 g/dl, serum albumin was 5.0 ± 0.3 g/dl, serum phosphate was 3.5 ± 0.2 g/dl, and plasma SID was 43.0 ± 2.4 mEq/L. Five aliquots were created for each subject by adding between 171 and 191 µl of the stock solutions. The resulting composition is reported in in Table 1 and Supplementary Figure S1, where it is evident that the shift in SID was achieved almost exclusively by manipulating [Cl^−^] or [Lac^−^], as desired.

**TABLE 1:** Characteristics of blood aliquots treated with the stock solutions. As most of the parameters change during the CO_2_ tonometry, values obtained by interpolation for pH = 7.2 are reported. Statistically significant difference from Ctr is marked by an asterisk (*).

		**Cl 15**	**Cl 7.5**	**Ctr**	**Lac 7.5**	**Lac 15**
BE(B)	mEq/L	−12.5 ± 2.1*	−6.2 ± 1.9*	0.6 ± 1.5	−6.0 ± 1.7*	−12.5 ± 2.1*
pCO_2_	mmHg	38.4 ± 7.4*	57.2 ± 7.0*	79.0 ± 5.8	57.5 ± 6.5*	38.3 ± 8.3*
[Na^+^]	mmol/L	146.0 ± 2.0*	145.4 ± 1.5*	144.7 ± 1.7	144.7 ± 2.0	144.5 ± 2.0
[K^+^]	mmol/L	4.2 ± 0.2*	4.0 ± 0.3	4.0 ± 0.3	4.0 ± 0.3	4.0 ± 0.3
[Ca^2+^]	mmol/L	1.30 ± 0.03*	1.28 ± 0.03*	1.25 ± 0.03	1.20 ± 0.02*	1.16 ± 0.03*
[Cl^−^]	mmol/L	120.1 ± 1.8*	111.8 ± 1.5*	103.1 ± 1.6	103.8 ± 1.5*	104.2 ± 1.4*
[Lac^−^]	mmol/L	1.5 ± 0.3	1.6 ± 0.4	1.5 ± 0.3	8.6 ± 0.4*	15.8 ± 0.7*
[HCO_3_^−^]	mmol/L	14.8 ± 2.7*	22.5 ± 2.5*	31.0 ± 2.2	22.6 ± 2.3*	14.8 ± 2.8*
SID	mEq/L	31.3 ± 3.5*	38.6 ± 3.0*	46.5 ± 2.8	38.7 ± 3.1*	30.8 ± 3.1*

CO_2_ tonometry of all aliquots was performed as described. The time elapsed between the first and last blood gas analysis of each aliquot was 14 [13–16] min, during which [Lac^−^] increased by 0.3 [0.1–0.5] mmol/L, and [K^+^] rose by 0.3 [0.3–0.4] mmol/L.

### β_NC_

**FIGURE 1:**
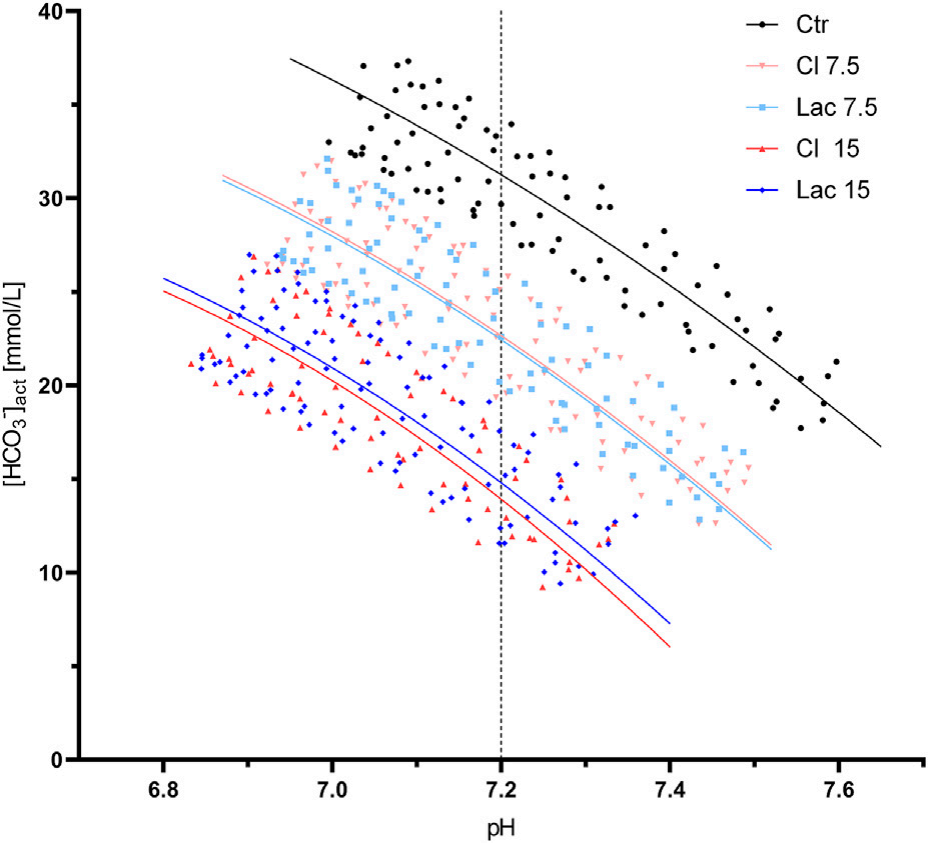
All valid pH/[HCO_3_^−^] data points obtained during the CO_2_ tonometry of the aliquots treated with the different stock solutions. Best-fit quadratic regression curve is shown for each group of aliquots. The pH of 7.2, at which β_NC_ was calculated, is marked by a dashed line.

Five aliquots (10%) were excluded from the analysis due to bad fit of the interpolated polynomial. All [HCO_3_^−^]/pH data points of the remaining aliquots are shown in Figure 1. Graphs that display data points of each volunteer separately and contain all the interpolated curves, including the eliminated ones, are presented in Supplementary Figure S2.

β_NC_, calculated at the pH of 7.2, was 28.0 ± 2.5 mmol/L in the control aliquots and increased to 30.6 ± 2.6 mmol/L and 33.8 ± 3.3 mmol/L in Cl 7.5 and Cl 15, respectively. Similarly, β_NC_ increased after the induction of experimental lactic acidosis to 30.4 ± 2.6 mmol/L and 33.5 ± 2.8 mmol/L for Lac 7.5 and Lac 15, respectively.

**FIGURE 2:**
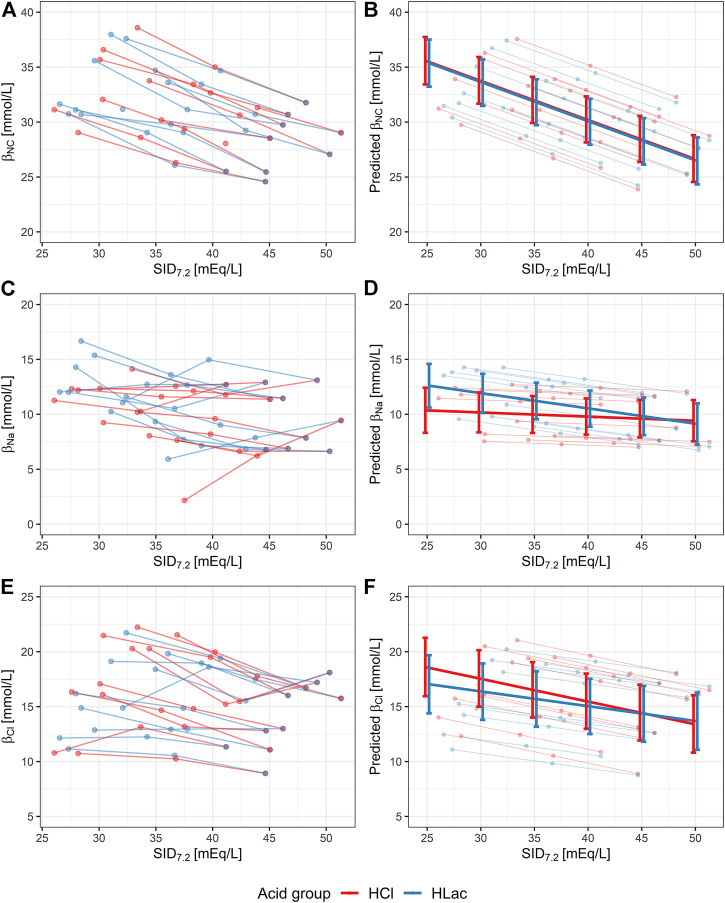
Noncarbonic buffer power (β_NC_) and non-carbonic buffer power due to change of [Na^+^] and [Cl^−^] (i.e., β_Na_ and β_Cl_) in aliquots with varying degrees of hyperchloremic (red) or lactic (blue) metabolic acidosis, characterized quantitatively by a reduction in SID_7.2_. The lines connect the aliquots treated with one type of acid and the control aliquot of each volunteer. Aliquots that were excluded due to bad fit of the interpolated curve are not shown. **(A,C and E)** Original data. **(B,D and F)** Results of linear mixed-effect model with random intercept (individual ID) showing 95% confidence intervals. SID_7.2_, acid type, and their interaction were considered fixed effects. In the case of β_NC_, the model was simplified by removing the fixed effect of interaction between the acid type and SID_7.2_.

Statistical significance of the observed change of β_NC_ was assessed using the described LMEM, for which the degree of metabolic acidosis was expressed quantitatively by means of SID_7.2_ (Figure 2A). The interaction between acid type and SID_7.2_ was not significant (*p* = 0.79). This means that the type of acid did not affect the slope of β_NC_ vs. SID_7.2_ and this interaction was removed from the model. The simplified model showed that the increase of β_NC_ in metabolic acidosis was significant (*p* < 0.001) with a rate of 0.36 mmol/L per 1 mEq/L decrease of SID_7.2_ and that the type of acid had no effect on β_NC_ (*p* = 0.37), regardless of SID_7.2_ (Figure 2B).

### The contribution of individual ions

The number of aliquots excluded from analysis due to bad fit was 1 for β_Na_, 1 for β_K_, 0 for β_Cl_, and 7 for β_Lac_. Over the studied pCO_2_ range (i.e., during the transition from 20 to 120 mmHg), [Na^+^] increased by 4.7 ± 1.3 mmol/L, [K^+^] increased by 0.1 ± 0.3 mmol/L, [Cl^−^] fell by 7.1 ± 1.7 mmol/L, and [Lac^−^] fell by 0.3 ± 0.3 mmol/L on average in all aliquots. In the control aliquots at the pH of 7.2, β_Na_ was 9.9 ± 2.7 mmol/L, β_K_ was 0.15 ± 0.57 mmol/L, β_Cl_ was 14.1 ± 3.1 mmol/L, and β_Lac_ was 0.24 ± 0.75 mmol/L.

The effect of metabolic acidosis, quantified using SID_7.2_, on β_Na_ and β_Cl_ is shown in Figures 2C,E. In both cases, the LMEM showed that the interaction between the acid type and SID_7.2_ was not significant (*p* = 0.09 and *p* = 0.07 for β_Na_ and β_Cl_, respectively). In other words, the slope of β_Na_ vs. SID_7.2_ and β_Cl_ vs. SID_7.2_ did not differ in the two types of acidosis. Nevertheless, we decided to keep this interaction in the model because of physiological plausibility of such observation and proximity of the *p* values to the threshold of significance.

According to LMEM, β_Na_ increased in lactic acidosis (*p* = 0.002, rate: 0.14 mmol/L per 1 mEq/L decrease of SID_7.2_) but did not change in hyperchloremic acidosis (*p* = 0.40), as shown in Figure 2D. β_Cl_ increased significantly both in hyperchloremic acidosis (*p* < 0.001, rate: 0.21 mmol/L per 1 mEq/L decrease of SID_7.2_) and lactic acidosis (*p* < 0.001, rate: 0.14 mmol/L per 1 mEq/L decrease of SID_7.2_), as shown in Figure 2F.

The changes in [K^+^] and [Lac^−^], that determine β_K_ and β_Lac_, are negligible and are not affected by either SID_7.2_, the type of acid, or their interaction (Supplementary Figure S3).

### The effect on BE(B)

Having demonstrated a significant increase of β_NC_ in metabolic acidosis, we proceeded to analyze its relevance for bedside acid-base diagnostics, i.e., the degree of bias it introduces into the calculation of BE(B). No aliquots were excluded from the following analyses.

**FIGURE 3:**
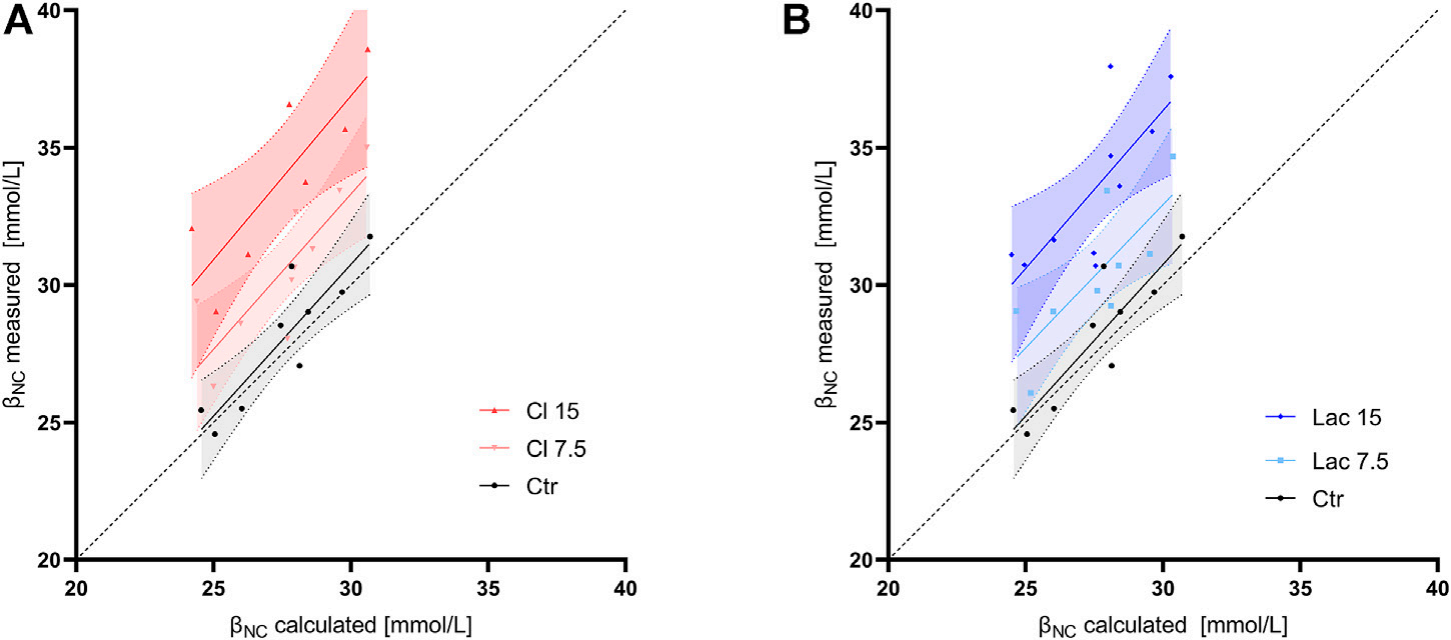
Noncarbonic buffer power calculated according to formula used in the Van Slyke equation (β_NC_ calculated) vs. the value measured using CO_2_ tonometry at a pH of 7.2 (β_NC_ measured). Linear regression line and its 95% confidence interval are shown for each group of aliquots. The dashed line is the line of identity. **(A)** The control aliquot and aliquots with induced hyperchloremic acidosis. **(B)** The control aliquot and aliquots with induced lactic acidosis.

**FIGURE 4:**
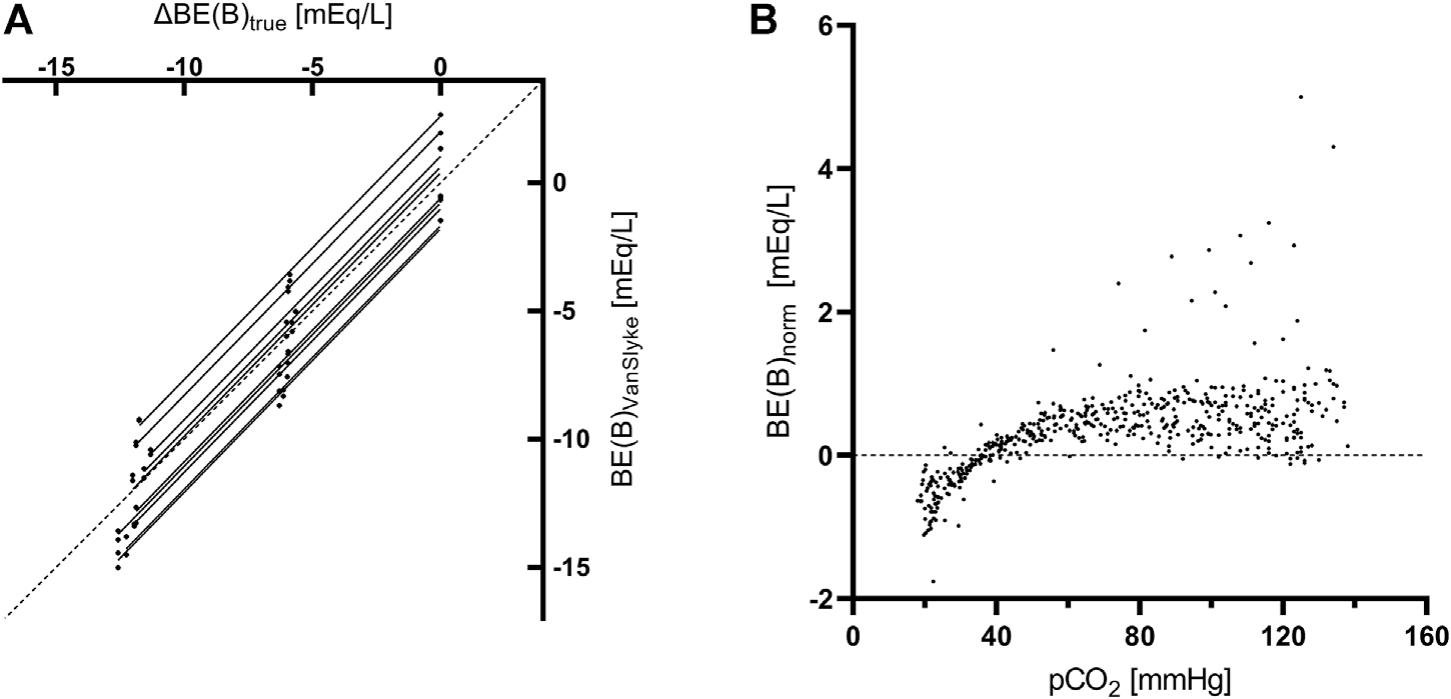
Analyses concerning the Base excess. **(A)** Linear regression of the true change of BE**(B)** determined from the amount and composition of the added stock solutions [ΔBE(B)_true_] vs. the value calculated using the Van Slyke equation [BE(B)_VanSlyke_] at pCO_2_ of 40 mmHg. Each aliquot is represented by 1 data point. The slope of the regression line was constrained as shared between volunteers and an individual regression line for aliquots of each volunteer is plotted. The dashed line is the line of identity. **(B)** Variance of normalized Base excess [BE(B)_norm_] due to pCO_2_ manipulations in whole blood. Each data point represents a single blood gas analysis.

In control aliquots, β_NC_ calculated according to the formula used in Van Slyke equation (Eq. 1) had a good agreement with the measured value at the pH of 7.2, while it systematically underestimated the measured β_NC_ in all samples with induced metabollic acidosis (Figure 3). The slope of the regression line, constrained as shared between volunteers, of BE(B)_VanSlyke_ vs. ΔBE(B)_true_ was 1.03 (95%CI: 1.01–1.05), i.e., slightly but significantly different from one (Figure 4A). The variation of BE(B) in response to pCO_2_ manipulation was small. The standard deviation of BE(B)_norm_ was only 0.64 mmol/L, and 93.8% of all blood-gas analyses were within the range of ±1 mmol/L from the BE(B) obtained at pCO_2_ of 40 mmHg (Figure 4B).

## Discussion

In this *in-vitro* study, we used CO_2_ tonometry to measure the non-carbonic buffer power of blood in healthy volunteers under normal conditions and after inducing experimental metabolic acidosis. Low [HCO_3_^−^], which accompanies metabolic acidosis, did not reduce the ability of blood to resist superimposed respiratory derangements. In fact, the exact opposite was true—β_NC_ was increased in the state of metabolic acidosis proportionally to its severity and irrespective of its type. The observed change of β_NC_ is in accordance with the theoretical prediction about the convergence of [HCO_3_^−^]/pH curves towards the acid side of the Davenport diagram (Siggaard-Andersen, 1974). This is because such convergence can only be observed if the curves representing pCO_2_ manipulation in metabolic acidosis, and thus positioned lower in the Davenport diagram, have a steeper slope and, therefore, higher β_NC_.

The action of intracellular non-carbonic buffers on plasma is possible due to transmembrane ion shifts: in response to the acidifying CO_2_ load, plasma SID increases mostly due to a reduction of [Cl^−^] in combination with a less prominent increase of [Na^+^]. In line, the Anion Exchanger 1, also known as Band 3 protein, responsible for Cl^−^/HCO_3_^−^ antiport, is the most abundant transport protein in the RBC membrane (Mohandas and Gallagher, 2008; Romero et al., 2013; Jennings, 2021). It has been shown that the RBC membrane is almost impermeable to cations and that RBC take up plasma water and swell in response to increasing CO_2_ tensions (Warburg, 1922; Van Slyke et al., 1923; Jackson and Nutt, 1954; Siggaard-Andersen, 1974). These observations imply, that transfer of solute-free water, rather than Na^+^, explain the observed change of [Na^+^]. Therefore, β_Na_ can be interpreted as a measure of free water transfer. We performed a post-hoc analysis using mean β_Na_, [Na^+^], and the estimated hematocrit of our samples to quantify the pCO_2_-induced change of hematocrit. Provided that β_Na_ was explained solely by free water transfer, the mean RBC volume decreased by 10% when pH rose by 1 unit due to pCO_2_ manipulation. This result agrees reasonably well with a theoretical prediction (Van Slyke et al., 1923)—see Figure 2 of the referenced article.

Our data show that the rise in β_NC_ is caused by an increased rate of ion and water transfer across the RBC membrane but do not provide a deeper insight into the underlying mechanism. We can hypothesize the following mechanisms or their combination:

• The allosteric effect of CO_2_, Cl^−^ or Lac^−^. The hemoglobin molecule is well known to interact with CO_2_, but binding of Cl^−^ and Lac^−^ has also been described (Guesnon et al., 1979). This mechanism could also explain the observed trend towards higher β_Cl_ in hyperchloremic acidosis and more pronounced β_Na_, i.e., water transfer, in lactic acidosis. Note that the allosteric effect of O_2_ was excluded by achieving high oxygen saturation at the beginning of CO_2_ tonometry.
• The active role of the RBC membrane. The permeability of the membrane to various charged species may limit the degree to which the intracellular buffers affect plasma. In other words, the properties of the RBC membrane may adjust the way the buffer action of hemoglobin is partitioned between the intracellular and extracellular fluid.
• The effect of intracellular pH. Different combinations of pCO_2_ and SID that we applied to achieve the pH of 7.2 in plasma, may have induced a different pH inside RBC, which is known to affect the intracellular non-carbonic buffer power (Goldsmith and Hilton, 1992).

The changes of plasma [K^+^] in response to acute disturbances of the acid-base equilibrium have been studied thoroughly, yet are far from being fully understood. Historically, blood pH was believed to be the sole determinant of the magnitude of transmembrane K^+^ shift. Later, it was found that these shifts are small or even non-existent in acute respiratory disorders. Moreover, it has become accepted that the [K^+^] change may differ markedly depending on the type of metabolic acidosis (Adrogué and Madias, 1981). Our results can contribute to this topic by showing that acute respiratory derangements did not induce any measurable transfer of K^+^ across the RBC membrane as β_K_, defined as -d[K^+^]/dpH during CO_2_ tonometry, was negligible (Supplementary Figure S3A,B). When it is clear that [K^+^] in our aliquots remained stable during pCO_2_ manipulation, it can be inferred from Table 1 that neither type of metabolic acidosis induced a clinically relevant change of [K^+^] despite the considerable rise of plasma [Cl^−^] or [Lac^−^] of up to 15 mmol/L. This is particularly remarkable for hyperchloremic acidosis, which is often used as an example of acidosis that triggers K^+^ release from the cells (Mount, 2022). This discrepancy may be due to the fact that the movement of K^+^ during acute acid-base disorders is not uniform among various tissues (Adrogué and Madias, 1981).

### Clinical implications

The observed changes in β_NC_ potentially have relevant clinical implications. First, the presence of metabolic acidosis improves the ability of non-carbonic buffers to mitigate the decrease of pH in response to a superimposed respiratory acidosis. This may represent a protective mechanism against encountering a fatally low pH value should a metabolic and respiratory acidosis occur simultaneously. It should be noted, however, that increased β_NC_ limits pH excursions in both directions equally. Therefore, the alkalizing effect of low pCO_2_, which defines the respiratory compensation, is attenuated as well.

Blood-gas analysis is a routine investigation in the acute care setting. In this context, β_NC_ is used by the blood-gas analyzers to calculate the Base excess, a key marker of the metabolic component of acid-base disorders. The automated calculation of BE(B), that derives β_NC_ value from hemoglobin concentration only, underestimated the actual β_NC_ value in both types of metabolic acidosis we studied. Despite that, BE(B) accurately reflected the amount of nonvolatile acid added. Although the slope of BE(B)_VanSlyke_ vs. ΔBE(B)_true_ did differ significantly from one, the observed difference is clinically irrelevant and, in fact, confirms the reliability of BE(B) calculation. The impact of β_NC_ on BE(B) calculation is, therefore, only minor, as suggested before (Langer et al., 2022). Importantly, BE(B) was independent of pCO_2_ to a great degree which is in line with similar tonometry experiments (Morgan et al., 2000).

We used BE(B) throughout this article as its definition matches our experimental setting of blood that is not equilibrated with the rest of the extracellular fluid. In clinical practice, however, the Base excess of extracellular fluid [BE(Ecf)], also known as Standard base excess (SBE), is a more precise parameter (Morgan, 2003; Langer et al., 2022). BE(Ecf) is computed similarly to BE(B) but uses a β_NC_ value that is determined from the estimated average hemoglobin concentration in the extracellular fluid. In healthy volunteers, β_NC_ for calculation of BE(Ecf) is, therefore, approximately one-third of the value used for BE(B). Consequently, the error introduced by variable β_NC_ into the Van Slyke equation, which was already very little during the calculation of BE(B), would be even smaller in the case of BE (Ecf).

## Limitations

The *in-vitro* nature of our study is clearly a limitation. While it allowed us to focus on buffering and exclude the confounding effects of other mechanisms that contribute to acid-base homeostasis (e.g., pulmonary and renal compensation), it prevented us from investigating the interaction between plasma and the rest of extracellular fluid, which is an important process *in-vivo*.

The action of intracellular buffers was only assessed using a proxy variable, meaning that the effect of pCO_2_ manipulation on intracellular -d[HCO_3_^−^]/dpH was not studied. Moreover, we neglected the possible binding of plasma ions to albumin (Halle and Lindman, 1978; Fogh-Andersen et al., 1993; Staempfli and Constable, 2003), assuming that the electrolyte and water shifts are the sole mechanism responsible for the observed changes of plasma electrolytes. Finally, we did not measure the hematocrit in relation to pH or pCO_2_ to confirm that β_Na_ represents electrolyte-free water shifts rather than transfers of Na^+^.

The sample size was rather small. Nevertheless, clear results were obtained for the main quantity of our interest, β_NC_, thanks to the repeated-measures design of our study and high precision of pH and pCO_2_ measurement.

Excluding aliquots from analysis due to bad fit of the interpolated curves could have limited the observed physiological variability. We believe, however, that the process was justified. In the case of β_NC_, three of the five eliminated aliquots represent a series of 31 consecutive blood-gas measurements during which the built-in quality control mechanisms of the analyzer detected abnormal results as well. All seven curves not included in β_Lac_ analysis belonged to the Lac 15 group. The usual precision of [Lac^−^] measurement was up to one decimal, but values higher than 15 mmol/L were rounded to the nearest integer. In some aliquots, this led to random oscillations between two values that did not represent the possible transfer of lactate across the RBC membrane.

## Conclusion

Blood aliquots with metabolic acidosis have an increased β_NC_, that, if confirmed *in-vivo*, may be an important mechanism that limits pH excursions caused by superimposed respiratory derangements. Although the formula employed in BE(B) calculation systematically underestimates β_NC_ in metabolic acidosis, this effect does not translate into BE(B) calculation in a way, that could be clinically relevant.

## Data Availability

The original contributions presented in the study are included in the article/Supplementary Material, further inquiries can be directed to the corresponding author.
