# Supplementary material for: Non-carbonic buffer power of whole blood is increased in experimental metabolic acidosis: An in-vitro study
Source: Front Physiol. 2022 Oct 21;13:1009378. doi: 10.3389/fphys.2022.1009378 (PMC9634561; doi:10.3389/fphys.2022.1009378)
Supplement: Supplementary file 2 [file DataSheet2.pdf]

# *Supplementary text*

## Part I: The transmembrane shift of electrolytes

Blood is several times more capable of resisting acid-base derangements than plasma (Ellison et al., 1958) thanks to the presence of powerful intracellular buffers, represented mainly by hemoglobin. However, when pH of blood is analyzed, the fluid in actual contact with the electrode is plasma (albeit in a close relationship with RBC). As plasma and hemoglobin are not in direct contact, the buffer action of hemoglobin on plasma can only be indirect, mediated by transmembrane shifts of electrolytes, as opposed to the direct buffer action of plasma weak acids.

The primary role of transmembrane electrolytes shifts as the means by which intracellular buffers act upon plasma can be easily demonstrated using Stewart's physicochemical approach (Stewart, 1981) to compare $CO_2$ titration of isolated plasma and the plasma phase of whole blood. Note that both fluids in this thought experiment consist only of a single compartment, which is necessary for applying Stewart's theory. According to this theory, pH of two fluids can only differ if at least one of the three independent variables ($pCO_2$, strong ion difference, or $A_{TOT}$) differs. Provided that the two studied fluids originate from the same donor and that plasma weak non-volatile acids (albumin, phosphates, and organic acids) do not cross the RBC membrane, their $A_{TOT}$ cannot differ. Neither $pCO_2$ can differ as it is the variable we manipulate. Therefore, the higher resilience of blood plasma to pH fluctuations, attributable to the presence of intracellular buffers, can only be mediated by alterations of its SID through transmembrane shifts of electrolytes.

Correspondingly, it has been shown in-silico that suppression of the chloride shift markedly decreases $\beta_{NC}$ of blood. At the same time, the usual difference between pH/[$HCO_3^-$] curves of oxygenated and deoxygenated blood, a footprint of the Haldane effect, disappears (O'Neill and Robbins, 2017). This implies that were it not for this electrolyte shift, the action of intracellular buffers would not be detectable in plasma.

## Part II: Hemolysis quantification

In preliminary experiments, hemolysis was quantified in 11 aliquots that underwent dilution and tonometry as described in Methods. The variables we analyzed were free plasma hemoglobin and [$K^+$] rise compared to fresh blood. The percentage of hemolyzed RBC was calculated assuming zero

free plasma hemoglobin in fresh blood and intracellular $[K^+]$ of 90 mmol/L (Beilin et al., 1966). It should be noted that $K^+$ is released from RBCs even without cell lysis and, therefore, the estimation of hemolysis by $[K^+]$ rise represents the upper limit of the hemolysis that could have occurred.

When manipulation with the aliquots was completed, free plasma hemoglobin was 0.13 [0.13-0.15] g/dL and plasma $[K^+]$ rose by 0.7 [0.6-1.0] mmol/L. The corresponding proportion of hemolyzed RBCs was 0.46 [0.45-0.53] % and 0.95 [0.81-1.36] %, respectively. According to the analytical standards, the amount of hemolysis we registered is classified as mild (Lippi, 2015).

**Part III: Accuracy analysis of the collected data**

For a given pH value, the ratio between the associated and dissociated form of plasma weak acids and, therefore, the amount of charge they expose is constant, determined by the difference between pH and $pK_a$ of each weak acid. Consequently, SID and $[HCO_3^-]$ obtained at the given pH level should correlate strongly in the five aliquots of each volunteer with the slope of the regression line equal to 1. As the calculation of SID and $[HCO_3^-]$ uses a different set of directly measured parameters ($[Na^+]$, $[K^+]$, $[Ca^{2+}]$, $[Cl^-]$, $[Lac^-]$ for SID; and pH, $pCO_2$ for $[HCO_3^-]$), exploring this correlation in our data can be regarded as a validation of its accuracy.

We defined $[HCO_3^-]_{7.2}$ in the same manner as $SID_{7.2}$ (see Methods) and explored the relationship between these two variables using linear regression with a shared slope and individual intercept for each volunteer.

Good agreement between $SID_{7.2}$ and $[HCO_3^-]_{7.2}$ was confirmed (Figure S4). The slope of the regression line shared between volunteers was 1.008 (95%CI: 0.974 to 1.043), i.e., not different from one (p=0.64). The mean difference between the two variables was -15.9±1.5 mEq/L, representing the net charge carried by plasma weak acids (albumin, phosphate, and organic acids) at pH=7.2 together with $Mg^{2+}$, which was not incorporated in our SID calculation. Thus, $[HCO_3^-]_{7.2}$ could have been used as the quantitative measure of the degree of metabolic acidosis in our study if we had chosen to use a bicarbonate-centered measure instead of $SID_{7.2}$.

**References**

Beilin, L. J., Knight, G. J., Munro-Faure, A. D., and Anderson, J. (1966). The sodium, potassium, and water contents of red blood cells of healthy human adults. *J. Clin. Invest.* 45, 1817–1825. doi: 10.1172/JCI105485.

Ellison, G., Straumfjord, J. V, and Hummel, J. P. (1958). Buffer Capacities of Human Blood and Plasma. *Clin. Chem.* 4, 452–461. doi: 10.1093/clinchem/4.6.452.

Lippi, G. (2015). "Systematic Assessment of the Hemolysis Index," in *Advances in Clinical Chemistry* (Elsevier Inc.), 157–170. doi: 10.1016/bs.acc.2015.05.002.

O'Neill, D. P., and Robbins, P. A. (2017). A mechanistic physicochemical model of carbon dioxide transport in blood. *J. Appl. Physiol.* 122, 283–295. doi: 10.1152/japplphysiol.00318.2016.

Stewart, P. A. (1981). *How to Understand Acid-Base*. New York: Elsevier.
