# Supplementary material for: Non-carbonic buffer power of whole blood is increased in experimental metabolic acidosis: An in-vitro study
Source: Front Physiol. 2022 Oct 21;13:1009378. doi: 10.3389/fphys.2022.1009378 (PMC9634561; doi:10.3389/fphys.2022.1009378)
Supplement: Supplementary file 3 [file Table1.pdf]

Table S1. The concentration of electrolytes in the stock solutions and their strong ion difference (SID).

		Cl 15	Cl 7.5	Ctr	Lac 7.5	Lac 15
[Na^+]	mmol/l	141	141	141	141	141
[Cl^-]	mmol/l	382	244	106	106	106
[Lac^-]	mmol/l	0	0	0	138	276
SID	mEq/l	-241	-103	35	-103	-241
