# Supplementary figures and images for: Non-carbonic buffer power of whole blood is increased in experimental metabolic acidosis: An in-vitro study
Source: Front Physiol. 2022 Oct 21;13:1009378. doi: 10.3389/fphys.2022.1009378 (PMC9634561; doi:10.3389/fphys.2022.1009378)

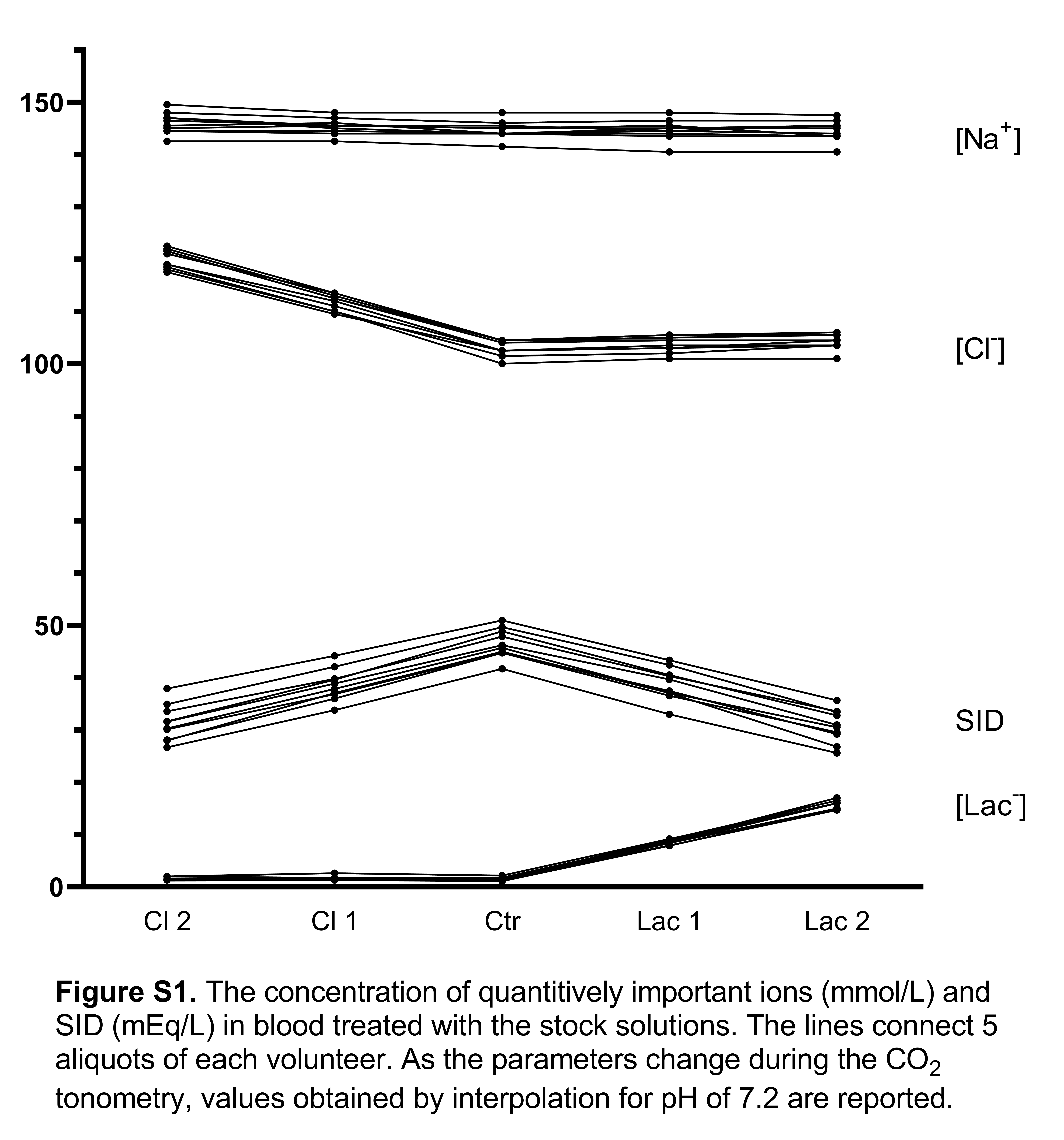

Supplement: Supplementary file 6 [file Image1.TIF]
